# Implementation of a program to support direct support professionals to promote a healthy lifestyle for people with moderate to profound intellectual disabilities

**DOI:** 10.1186/s12913-021-07389-x

**Published:** 2022-01-02

**Authors:** A. Overwijk, T. I. M. Hilgenkamp, C. P. van der Schans, W. P. Krijnen, K. Vlot-van Anrooij, A. A. J. van der Putten, A. Waninge

**Affiliations:** 1 Research Group Healthy Ageing, Allied Health Care and Nursing, Hanze University of Applied Sciences Groningen, Groningen, the Netherlands; 2 Department of Health Psychology, University of Groningen, University Medical Center Groningen, Groningen, the Netherlands; 3 Department of General Practice, Intellectual Disability Medicine, Erasmus MC, University Medical Center Rotterdam, Rotterdam, the Netherlands; 4 Department of Physical Therapy, University of Nevada, Las Vegas, USA; 5 Department of Rehabilitation Medicine, University of Groningen, University Medical Center Groningen, Groningen, the Netherlands; 6 Department of Primary and Community Care, Research group Intellectual Disabilities and Health, Radboud Institute of Health Sciences, Radboud University Medical Center, Nijmegen, the Netherlands; 7 Department of Inclusive and Special Needs Education, University of Groningen, Groningen, the Netherlands

**Keywords:** Health education, Health promotion, Implementation, Attitude, Behaviour, Caregivers, People with ID

## Abstract

**Background:**

There is a lack of theory-based interventions for direct support professionals (DSPs) to support a healthy lifestyle for people with moderate to profound intellectual disabilities (ID) despite their major role in this. This study aims to evaluate the preparation, implementation, and preliminary outcomes of a theory-based training and education program for DSPs to learn how to support these individuals.

**Methods:**

The program consisting of e-learning, three in-person sessions, and three assignments was implemented. The implementation process was evaluated with a mixed method design with the following components: preparation phase, implementation phase, and the outcomes. These components were measured with project notes, questionnaires, interviews, reflections, assignments, food diaries, Actigraph/Actiwatch, and an inventory of daily activities.

**Results:**

Regarding the preparation phase, enough potential participants met the inclusion criteria and the time to recruit the participants was 9 months. The program was implemented in four (residential) facilities and involved individuals with moderate to profound ID (*n* = 24) and DSPs (*n* = 32). The e-learning was completed by 81% of the DSPs, 72–88% attended the in-person sessions, and 34–47% completed the assignments. Overall, the fidelity of the program was good. DSPs would recommend the program, although they were either negative or positive about the time investment. Mutual agreement on expectations were important for the acceptability and suitability of the program. For the outcomes, the goals of the program were achieved, and the attitudes of DSPs towards a healthy lifestyle were improved after 3 months of the program (nutrition: *p* = < 0.01; physical activity: *p* = 0.04). A statistically significant improvement was found for food intake of people with ID (*p* = 0.047); for physical activity, no statistically significant differences were determined.

**Conclusions:**

The theory-based program consisting of a training and education section for DSPs to support a healthy lifestyle for people with moderate to profound ID was feasible to implement and, despite some barriers regarding time capacity and mutual expectations, it delivered positive changes in both persons with moderate to profound ID and DSPs. Thus, the program is a promising intervention to support DSPs.

## Background

People with moderate to profound intellectual disabilities (ID) often do not have a healthy lifestyle [1–4] with regard to physical activity and healthy nutrition [5, 6]. These individuals often have low levels of physical activities and an unhealthy diet [2, 6]. Consequently, people with moderate to profound ID have a higher prevalence for health problems like constipation and being under- or overweight [7]. Severe motor disabilities as an example of prevalent health problems are also barriers for physical activity participation of people with ID. Other barriers are related to individual motivation and preferences; support on financial level, transportation and staffing levels. Facilitators for physical activity are for example social interaction and engagement, rewarding’s, and having fun [8]. People with moderate to profound ID require support from their social environment in performing their daily activities and thus to live healthily. In the Netherlands, this support is often provided by direct support professionals (DSPs) in residential facilities. Therefore, DSPs play a major role in the support of a healthy lifestyle for people with moderate to profound ID [9, 10]. These DSPs usually have an educational background in social work or nursing for which a healthy lifestyle is not an element of the program. However, training and education can help DSPs to support a healthy lifestyle [11].

To support a healthy lifestyle, there are theory-based motor activity programs for people with profound intellectual and multiple disabilities [12, 13]. However, previous research shows a lack of theory-based interventions and education for DSPs to support a healthy lifestyle [14]. Theory-based interventions are shown to have more potential for effective outcomes than interventions without a theoretical basis [15]. In a previous study, a theory-based training and education program was developed in co-creation with daily practice [16]. The content of the training and education program is based on the Theoretical Domains Framework (TDF), related to the COM-B System [17, 18], and Behaviour Change Techniques (BCTs) [19]. The TDF gives insights about the conditions to support a healthy lifestyle. The COM-B system is complementary to the TDF whereas this system explains the nature of behaviour to change it. In addition, one of the skills DSPs need is motivating people with ID towards healthy behaviour, therefore BCTs are added to the program. The mode of delivery of the program was based on Kolb’s theory of Learning Styles [20]. The developed program consists of a training and education section with online and in-person components to support DSPs in promoting healthy living for people with moderate to profound ID. In the online component, DSPs gather knowledge and increase awareness of physical activity and healthy nutrition for this population. For the in-person component, DSPs participate in three sessions to discuss health promotion with the following themes: Social/Professional Role and Identity, Skills (Behaviour Change Techniques), and Social Influences, Environmental Context and Resources.

A next step is to examine the potential of this intervention by implementing the program and evaluating this process [21]. However, the implementation of newly developed programs has been shown to be a major challenge in organizations in general [22] and for ID care providers [11, 12, 23]. Reporting of implementation processes and identifying key facilitators and barriers is important for compiling a knowledge base for successful implementation [22]. ID care providers and researchers can learn from previously implemented interventions and apply that knowledge to advance implementation processes [12, 23]. In order to evaluate its potential for widespread implementation, the aim of this study is to evaluate the preparation, implementation, and preliminary outcomes of a theory-based training and education program for DSPs to learn how to support people with moderate to profound ID in a healthy lifestyle.

## Methods

### Design

**Fig. 1:**
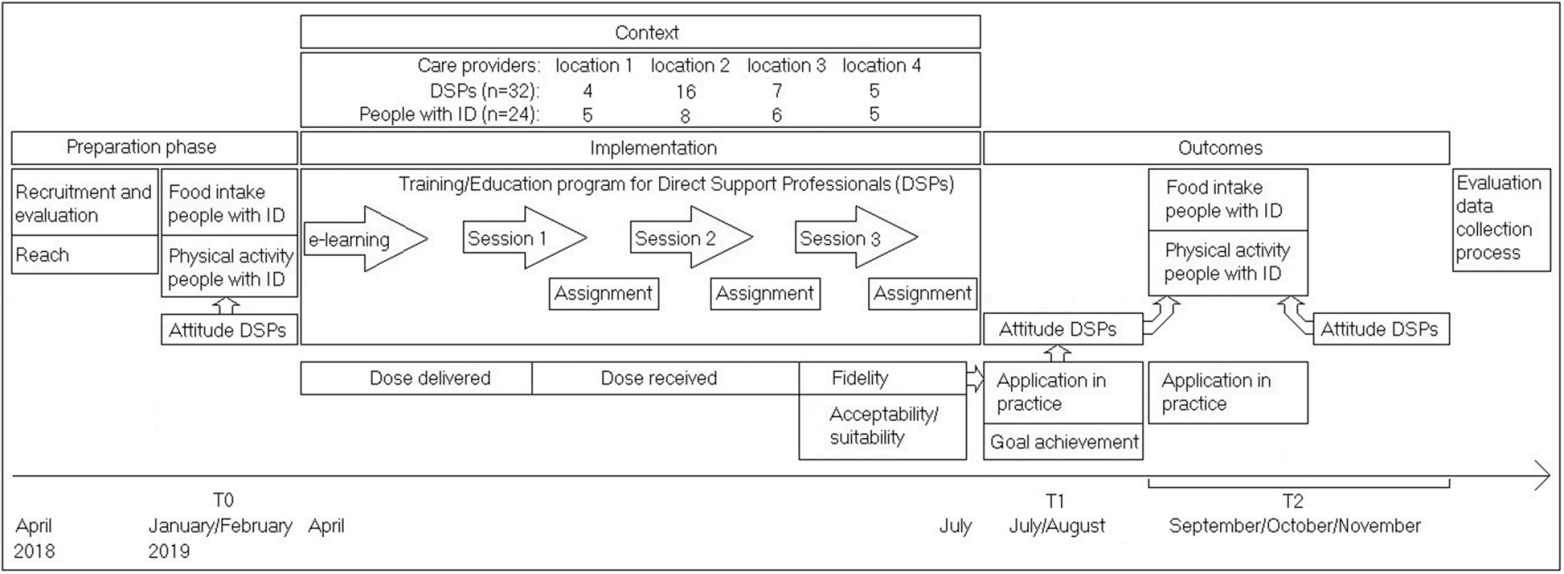
Design of the study

A theory-based program consisting of a training and education section to facilitate DSPs in promoting a healthy lifestyle for people with moderate to profound ID was implemented in four (residential) facilities of ID care providers in the Netherlands. A mixed method design was used to evaluate the implementation phase. This implementation process was operationalised and evaluated with the following components: recruitment, reach, context, dose delivered, dose received, fidelity [24], recruitment capacity, acceptability/suitability of the program, factors during implementation, data collection process, and changes in both DSPs and persons with ID after the program [25]. Figure 1 illustrates the design of the study.

### Sample and context

The participants of four residential facilities and/or day activity centres from four different ID care providers were recruited within a consortium of eight ID care providers across the Netherlands. The inclusion criteria for the (residential) facilities were as follows:

- A (residential) facility where people with moderate to profound ID (≥18 years) receive support.
  In this study people with moderate to profound ID are defined as individuals who need support in several domains, for example in language, motor skills, sensory, and in activities for daily living [26, 27]. These people experience limitations in both intellectual and adaptive functioning, in the following domains: conceptual, social, and practical adaptive skills. In addition, people with more severe intellectual disabilities experience more often mobility problems [28]. Based on the support needs people with ID are categorized as having moderate, severe, or profound ID.
- Readiness for implementation of the (residential) facility by checking the fit of the goal of the program in consultation with the facility.
- Availability of one trainer to support the program.
- Time capacity of the team for participating in the research study by receiving approval from the manager for the indicated time investment. .

The contact persons of the ID care providers assisted in the recruitment of the teams working at the selected (residential) facilities by informing in person and distributing information flyers. If the team was interested in participating, the researchers contacted the team to discuss participation. If the recruited team had the capacity to participate, the DSPs, people with ID, and legal representatives were informed and asked for consent for the study.

The Discovering Health-promoting Assets in Settings for people with Intellectual Disabilities (DIHASID) tool was used to gain insight in the implementation context [29]. The DIHASID tool provides actionable knowledge about the social, physical, financial, and organizational assets for physical activity and healthy nutrition at a residential or day-activity accommodation [30]. From the enquired physical assets, seven out of 13 were available at all (residential) facilities, and an additional three were available at three (residential) facilities. For example, there was enough space at all of the (residential) facilities for physical activities. Some aids and equipment, such as activity-stimulating games, were not available for all settings. At all (residential) facilities, social assets were available, for example, health professionals to help with exercise activities. However, to support healthy living, friends, clients, volunteers, and a massage therapist were accessible at only one or two (residential) facilities. At the policy level, the focus on healthy living in organisations was moderate to satisfactory; financial assets were also moderate to satisfactory [31].

### Theory-based program

A theory-based program for DSPs to learn how to support physical activity and healthy nutrition for people with moderate to profound ID was implemented [16]. The program consisted of (see also Fig. 1):

1. E-learning to increase knowledge and awareness of physical activity and healthy nutrition for people with moderate to profound ID.
2. Three in-person group sessions of 2 h with the following themes:
  Social/Professional Role and Identity.
  Skills (Behaviour Change Techniques, BCTs).
  Social Influences and Environmental Context and Resources.

The e-learning could be performed individually or with colleagues; the in-person sessions were performed within the team of DSPs at the (residential) facility where they work. Between and after the last in-person group session, practical assignments (three in total) were carried out within the team of DSPs.

The program was in operation from April 2019 to July 2019. The training was performed by two trainers: one trainer from the Hanze University of Applied Sciences (an experienced teacher from the social work program) and one trainer of each ID care provider (e.g. a behaviour scientist). Prior to the beginning of the program, the trainers received an instruction manual including PowerPoint presentations in order to prepare for the in-person sessions. A common meeting with the trainers was held to coordinate the sessions and to clarify uncertainties. After each session, there was brief contact with the trainers and with the first author about the process of the program. The e-learning was implemented in collaboration with the technical staff of the involved care providers. Before and during the program, there was consultation with the managers regarding the implementation (e.g. manager Knowledge and Innovation as well as manager residential facilities and day activity centers) and contact persons of the involved ID care providers in order to improve the implementation phase.

### Data collection

To measure the implementation components, the definitions and underlying questions of Linnan and Steckler [24] and the objectives of Orsmond and Cohn [25] were used. The methods for data collection can be found in Table 1; the design of the study is depicted in Fig. 1. In the preparation phase, for the evaluation of recruitment and reach, project notes were captured. For the changes in the context, questionnaires and interviews were used.

**Table 1:** Components of implementation, indicators, data collection and analysis

Component	Indicator	Data collection	Analysis
**Preparation phase**			
Recruitment and evaluation	-Recruitment process -Participant number -Time to recruit -Refusal rates -Feasibility/suitability inclusion criteria -Obstacles recruitment -Relevance of program to population	-Project notes	-Document analysis [32]
Reach	Number of DSPs from team	Project notes during delivery	-Calculation of DSPs from team
Context	-Environment changes (physical, social, political) -Other interventions	-Questionnaire DSPs -Questionnaire managers -Interviews DSPs	-Check on changes in environment beside the program -Check on other interventions (yes/no)
**Implementation phase**			
Dose delivered	Completion program	Project notes during delivery	-Calculation of completion of each part of the program
Dose received	-Execution of assignments -Activity during sessions	-Questionnaire DSPs -Reflection of trainers on sessions -Reflection of researcher (AO) on one session -Quality of assignments	-Descriptive statistics -Quality assignments checked with the following question: ‘Do the assignments show that the DSP understood the assignment?’ [23], and scored according to the following categories: ++ Almost everything on the assignment is correct, + there are more points on the assignment that are correct than points that are not correct, − there are fewer points on the assignment that are correct than points that do not match, -- almost everything on the assignment is not correct.
Fidelity	Execution of sessions by trainers	-Reflection of trainers on sessions -Reflection of researcher (AO) on one session	-Descriptive statistics
Acceptability/suitability program	-Fit of program in daily practice (trainers, DSPs, managers) -Acceptability of program for DSPs and managers -Time/capacity to complete program (DSPs, managers)	-Reflection of trainers on sessions -Questionnaire DSPs -Questionnaire managers -Interviews DSPs	-Document analysis -Descriptive statistics -Conventional content analysis [33]
Factors during implementation	-Facilitators/barriers during implementation (expertise, capacity, budget, equipment available)	-Project notes during implementation	-Document analysis
Evaluation of data collection process	-Feasibility/suitability data collection procedures (missing data, understanding questions, time-consuming) -Sensitivity of outcome measures to changes after the program	-Project notes during data collection -Evaluation of outcomes in relation to methods	-Document analysis
**Outcomes**			
Changes after the program	-Goal achievement after program -Actual application in practice (DSPs, assignments, managers) -Attitude DSPs (T0, T1 and T2) -Food intake people with ID (T0, T2) -Physical activity of people with ID (T0, T2)	-Questionnaire DSPs -Questionnaire managers -Interviews DSPs -Assignments -Food diaries -Actigraph/Actiwatch/ Inventory of daily activity program	-Descriptive statistics -Conventional content analysis [33] -Application check assignments -Attitude changes: linear mixed models [34] in statistical programming language R [35]. -Food diaries: descriptive statistics, Wilcoxon signed Rank test -Actigraph: descriptive statistics, Wilcoxon signed Rank test -Actiwatch: descriptive statistics, calculating %inactive/active time -Inventory of daily activity program: descriptive statistics, Wilcoxon signed Rank test

In the implementation phase, the dose delivered, dose received, and fidelity were captured with project notes, questionnaires, reflections, and assignments. The acceptability/suitability of the program was measured with reflections, questionnaires, and interviews. The last parts of the implementation phase, the factors during implementation, and the evaluation of the data collection process were measured with project notes. During the implementation phase of the program, the first author was present at one in-person session of the program with each of the four participating teams in order to observe whether the sessions were carried out as intended.

For the preliminary outcomes, the changes after the program for DSPs were measured in terms of goal achievement, actual application in practice, and attitude of DSPs towards nutrition and physical activity. These data were aggregated from questionnaires, interviews, and assignments. Goal achievement was measured with a questionnaire for DSPs 1 week after the last program session. The actual application of healthy lifestyle support in daily practice was measured during the program with practical assignments for DSPs and 3 months after the program with interviews with them. Managers were also surveyed about the actual application of the program in daily practice. The attitude was defined as the thoughts and feelings of DSPs regarding a healthy lifestyle [11]. The attitude of DSPs towards physical activity and healthy nutrition was measured before the program (T0), 1 week after (T1), and 3 months after (T2) the last program session [11]. The attitude was measured on a 5-point Likert scale for which 1 indicates a negative and 5 a positive attitude. The psychometric properties of the questionnaires were favourable [11] (Overwijk A, Krijnen WP, Hilgenkamp TIM, Van der Schans CP, Van der Putten AAJ, Waninge A: A questionnaire to measure direct support professionals’ attitude towards healthy nutrition of people with intellectual disabilities, submitted).

To evaluate the program, individual telephone interviews were held with half of the participating DSPs (*n* = 17) as a sufficient representation on the following topics: recommendation of the program, connection to support needs, actual implementation in daily practice, and other interventions during the program. In addition, changes in the level of physical activity and the amount of food intake of the participating people with ID were measured. Physical activity was measured with the Actigraph wGT3X BT for walking respondents [36, 37] and with the Actiwatch for non-walking respondents [38]. The data for physical activity of the walking people with moderate to profound ID was collected with a frequency of 30 Hz [37], over 1 min epoch periods [39]. Additionally, DSPs recorded the planned movement activities during the measurements of physical activity. Food intake was measured with food diaries covering 3 days [40]. Food intake and physical activity were measured before (T0) and 3 months after (T2) the last program session.

### Data analyses

In the last column of Table 1, details regarding the analyses are described. In the preparation phase, the project notes for the evaluation of recruitment and reach were analyzed with documentary analyses [32], and the DSPs per team were calculated. For the changes in the context, questionnaires and interviews were checked in other interventions (yes/no) and influential changes in the environment were examined.

In the implementation phase, the dose that was delivered was calculated based on the completion of the e-learning, in-person sessions, and the assignments. For the dose received, descriptive statistics were used, and the quality of assignments was checked, and they were scored for correctness [23]. For the fidelity of the program, the descriptive statistics for the reflections of the trainers and the researcher (AO) were analyzed. The acceptability and suitability of the program was analyzed with a document analysis in order to determine the reflection of the trainers regarding the open questions about the learning questions of the teams and additional comments. Descriptive statistics were used to analyze the questionnaires of DSPs and managers. In addition, a conventional content analysis [33] using ATLAS.ti was employed to analyze the suitability and acceptability of the program for DSPs. The factors during implementation and the evaluation of the data collection process was analyzed with a document analysis of the project notes made during the implementation.

For the preliminary outcomes, the changes after the program were analyzed with descriptive statistics for goal achievement. The actual application in practice was analyzed with a conventional content analysis [33], and the application was checked in the assignments. The changes in attitudes of DSPs towards a healthy lifestyle were analyzed with linear mixed models [34] in the statistical programming language R [35].

To analyze the changes after the program for people with ID, data for the food intake of people with ID were compared with the Dutch nutrition guidelines via a food meter. This is a Dutch tool to support appropriate composition of food intake according to the recommended nutrition guidelines. Of the food intake, 85% should meet the nutrition guidelines [41]. For physical activity, the cutoff points of Freedson et al. [42] were used for the Actigraph data according to Chow et al. [39]. Non-wear time was defined as 60 min, and a minimum wear time per day was set for 600 min with a minimum of 4 days of valid wear time. Only the non-wear time was excluded from analysis. The low frequency filter (LFE) was checked for low sensitive movements. The cutoff points of Van Alphen et al. [38] were used for the respondents wearing the Actiwatch; 0–15 counts inactive, > 15 counts active. The data regarding both physical activity and nutrition before (T0) and 3 months after (T2) the program were compared using the Wilcoxon signed rank test. For the Actiwatch data, the percentages of inactive and active time were calculated.

To obtain insight into the differences between the participating (residential) facilities and the outcome variables for DSPs (goal achievement, changes after program, relevance program, satisfaction program, and attitude) and people with ID (food intake, and physical activity), a one-way ANOVA was performed.

Two trained master students of Inclusive and Special Needs Education supported analysing the interview data with a conventional content analysis [33] and the food diaries, and one bachelor student of human movement sciences supported with the entry of the food diaries.

## Results

The results are described in the order of the implementation process (see Table 1). The preparation phase will be reported first, and then the implementation phase will be discussed and, lastly, the outcomes with corresponding variables will be examined.

### Preparation phase

#### Evaluation of recruitment and reach

Four residential facilities and/or day activity centres supporting adults with moderate to profound ID were included. All of the approached teams were enthusiastic about the program and its relevance. The time to recruit the appropriate number of participants was 9 months (April 2018 until January 2019). DSPs also spent time to inform and obtain informed consent of the people with ID. The inclusion criteria could be met for this study, although difficulties were faced, for example, if people with mild ID lived or worked together with people with moderate to profound ID, and thus the target group did not completely match. In addition, the required time investment of the DSPs for the program and for the data collection was a challenge for the (residential) facilities.

Participants in this study were DSPs (*n* = 32), people with moderate to profound ID (*n* = 24), managers/coordinator of the participating (residential) facilities (*n* = 4), and the trainers of the program (*n* = 6). In total, 32 DSPs out of 41 DSPs from the four (residential) facilities participated in the study. The distribution of DSPs over the four (residential) facilities was as follows: 4, 16, 7, and 5 DSPs. Reasons for not doing so were: lack of presence at the (residential) facility at the time of the implementation due to attendance in another department of the ID care provider, not willing to commit to the time investment, or too recently hired. The characteristics of the DSPs and the people with moderate to profound ID supported by the participating teams are described in Table 2.

**Table 2:** Characteristics of the DSPs and people with ID

**DSPs (*n*=32)**	
Age in years, mean (SD)	34 (11)
Gender female, n	25
Education, n	
Senior secondary vocational education: Educational theory	11
Senior secondary vocational education: Nursing	2
University of applied sciences: Educational theory	12
University	1
Other:	6
Supplemental lifestyle training, Yes	7
Work setting, n	
Residential facility	11
Day activity centre	5
Combination group	16
Years of experience with people with ID, mean (SD)	11 (10)
Years working on current workplace, mean (SD)	3 (3)
DSP-to-people with ID ratio, mean (SD)	5 (4): 11 (7)
**People with ID (*n*=24)**	
Age in years, mean (SD)	38 (17)
Gender female, n	8
Degree of ID, n	
Moderate ID	9
Severe ID	8
Profound ID	7
Wheelchair-user, n	3

#### Context

During the program, there were changes in the context that were not provoked by the implementation of the program. These changes were with regard to staff: decrease in staff at one (residential) facility (number 1) and additional education for two DSPs at one (residential) facility in order to become physical activity consultants (number 2). With regard to a healthy lifestyle, the following changes were made: decrease in budget for food at one (residential) facility (number 2), increasing offer of physical activity at one (residential) facility (number 4), and health related activities at two (residential) facilities (numbers 1 and 4), for example, receiving recommendations from a movement expert regarding physical activity, more indoor activities, a short class for people with ID about health, and a cooking activity 1 day per week for people with ID.

### Implementation phase

#### Dose delivered, dose received and fidelity

All program components (e-learning, three in-person sessions, and three assignments) were available, although not all DSPs completed all of the components of the program. The e-learning was completed by 26 out of the 32 DSPs. All of the three in-person sessions were attended by 18 DSPs. The remaining 14 DSPs attended one or two in-person sessions. Reasons for not participating in the in-person sessions were illness, vacation, time, or private circumstances. An overview of dose delivered and dose received is described in Table 3. With regards to completing the assignments, six DSPs completed all three assignments, 13 completed either one or two assignments, and 13 did not complete any assignments.

**Table 3:** Dose delivered and dose received

	Dose delivered (*n* = 32), n	Dose received (*n* = 32), n (%)
E-learning	32	26 (81)
In-person session 1	32	28 (88)
Assignment 1	32	15 (47)
In-person session 2	32	28 (88)
Assignment 2	32	12 (38)
In-person session 3	32	23 (72)
Assignment 3	32	11 (34)

The quality of the submitted assignments (38 out of 96) was mostly positive (23 assignments); see Table 4. The quality of the assignments was considered not sufficient if not all parts of the assignment were completed (e.g. there was no consultation with colleagues), the link to the theme of the in-person session was missing, a question was not well understood, or the BCTs were not clear enough.

**Table 4:**
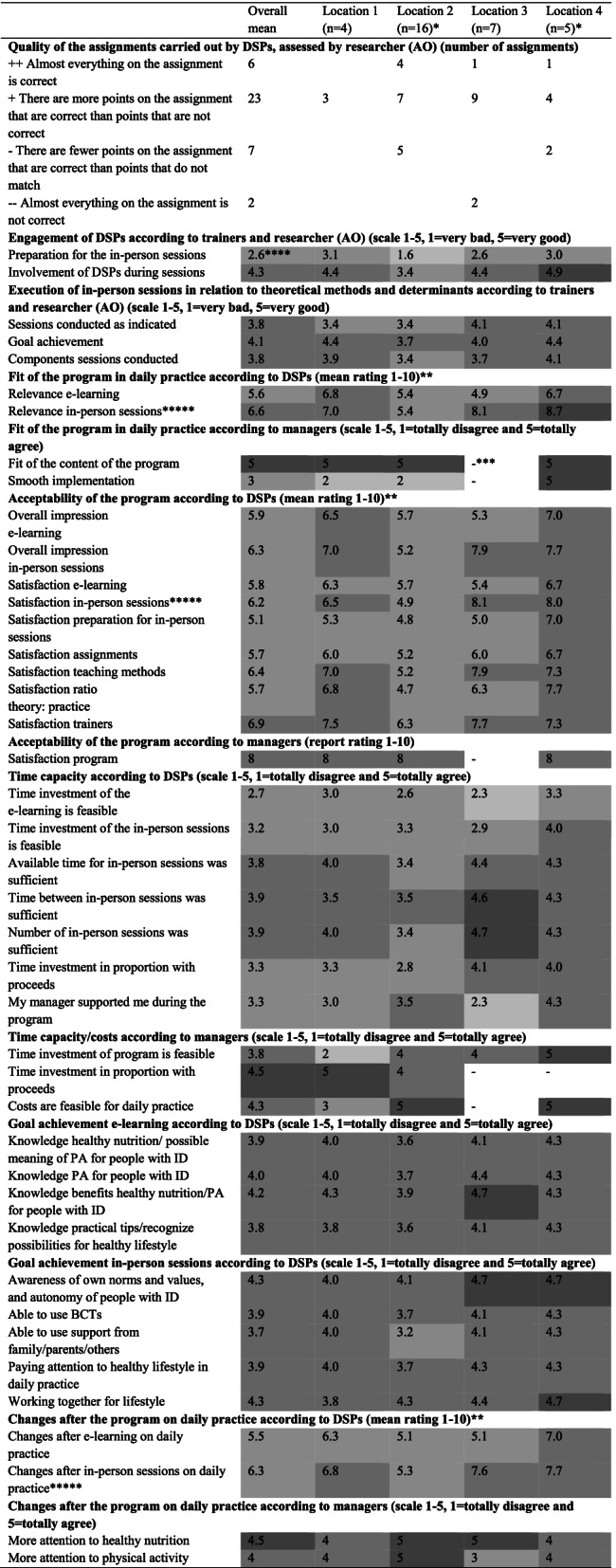
Results of implementation: dose received, fidelity scores, acceptability and suitability of the program, and changes after the program (goal achievement, actual application in practice)

^a^One DSP did not fill in the complete questionnaire

^b^For (residential) facility 4 three DSPs answered the questions

^c^This question could not be answered

^d^The grey colour in the cells represent negative, neutral or positive results: the darker the colour the more positive. For the rating: 1–2 is very negative, and 9–10 is very positive

^e^Statistically significant differences between (residential) facilities

According to the DSPs, 13 out of 32 prepared all of the in-person sessions. Some DSPs prepared only partly for the sessions. Reasons for not preparing were illness, absence, time, and not remembered. The trainers indicated that DSPs from two (residential) facilities in particular prepared inadequately for the in-person sessions. However, the participation during the sessions at these two (residential) facilities was neutral to good. One (residential) facility had different expectations (working with more clear action points) for the in-person sessions. Additionally, the group size for one (residential) facility was large (*n* = 16), making the sessions less effective according to the reflection of the trainers. Overall, the execution of the in-person sessions in relation to theoretical methods and determinants was good (see Table 4).

#### Acceptability and suitability of the program

Table 4 shows the mean ratings of DSPs and the managers for the acceptability and fit of the program. It also indicates that, for two of the four (residential) facilities, the acceptability of the program was sufficient, and the fit of the program in daily practice was sufficient to good according to DSPs. For the other two (residential) facilities, the acceptability and suitability were insufficient to good; for one (residential) facility, the acceptability and relevance of the e-learning was considered insufficient whereas the satisfaction and relevance of the in-person sessions were good. The overall satisfaction about the preparation before the in-person sessions was insufficient according to DSPs. The satisfaction and the relevance of the in-person sessions was, for (residential) facility 2, statistically significantly lower in comparison to the other three (residential) facilities. The DSPs of (residential) facility 2 were overall less satisfied about the program and its relevance. All managers granted the program an eight as a rating (one manager could not answer this question), and three out of four were very positive about the content of the program (missing information for one manager).

Almost all of the interviewed DSPs (14 out of 17) indicated that they would recommend the program to colleagues. The DSPs mentioned that their recommendation depends on the status of the team in the domain of healthy lifestyle. Comments that were more critical by some of DSPs pertained to the lack of connection with the target group and the significant time investment of the program. In addition, all of the interviewed DSPs indicated that they were positive (*n* = 17) about the connection of the program to their support needs. Approximately half of them (*n* = 8) considered the awareness of a healthy lifestyle and communicating with each other in this context as positive. One DSP illustrates: ‘*I thought it was good. We do a lot of things, but it is the awareness of just simple things, like housekeeping, that is also physical activity. Often, we take over activities of our clients which they can do by themselves’* (respondent 5:9). Beside the positive statements about the connection of the program to support needs, there were also DSPs who made neutral (n = 8) or negative (*n* = 7) comments. They indicated that the program was not necessary or that they expected more tips for daily practice.

#### Time and capacity to complete the program

Per DSP, the time investment of the program, including preparation and assignments, was an average of 11 h. The feasibility of the time investment of the e-learning was, according to the DSPs, slightly negative to neutral. The feasibility of the time investment of the in-person sessions was neutral to positive. They indicated that the number of sessions, the available time, and the time between the in-person sessions was good. The time investment of the program and the proceeds were rated neutral to positive. The experienced support from their manager was slightly negative to positive according to the DSPs (see Table 4 for their ratings). Overall, the managers indicated the time investment of the program was feasible with the exception of a single manager indicating that the time investment was too much, especially for the research element. The costs associated with the time investment were feasible according to the managers. They were positive about the time spent on the program and the outcomes (see Table 4 for the ratings of the managers). The capacity to complete the program as operationalized by DSPs’ participation in the activities during the meetings was sufficient (score 4.1 on a scale from 1 to 5).

#### Factors during implementation

Factors for implementation were addressed when developing the program and designing the study, for example, continuous communication about the program [16]. The primary researcher was able to manage the conditions to facilitate the implementation of the study and the program. The willingness to participate was good, however, despite careful discussions of expectations, the time for the DSPs to participate in the study and the program was limited. In addition, according to two of the four managers, the implementation of the program requires improvement, for example. With respect to the alignment of mutual expectations before the beginning of the program.

#### Evaluation of the data collection process

Based on the goals of the program, the overall sensitivity of the outcome measures to the changes after the program was good. The DSPs understood the questions and guidelines to complete the data collection. They were requested to fill in questionnaires, participate in interviews, and track food intake and physical activities of people with ID. This data collection process appeared, as noted before, time consuming for the participating teams which resulted in missing data. Regarding the missing data for the food diaries, at T2, 6 days of six people with ID were missing. Additionally, part of the diaries or details were missing which could not be included. For physical activity at T0, six people with ID did not wear the Actigraph for at least 4 days for 10 h, and one of them lost the Actigraph. Additionally, at T2, three people with ID did not wear the Actigraph.

### Preliminary outcomes

#### Changes after the program

Figure 1 and Table 1 provide an overview of the design and the indicators for the changes after the program. The outcomes will be described based on the indicators: goal achievement of the program, actual application in practice, attitude of the DSPs, food intake of people with ID, and physical activity of people with ID.

#### Improving knowledge and skills

Overall, the DSPs’ goals of the program have been achieved for both the e-learning and the in-person sessions; see Table 4. They rated their knowledge about physical activity and nutrition for people with ID; these ratings were a 6.6 and a 7.2, respectively, at T0 and T1. This shows an improvement in knowledge directly after the program (Z = -2.923, *p* = 0.003).

#### Actual application in practice

The change in daily practice following the program was sufficient to good for two of the four (residential) facilities according to DSPs. For one (residential) facility, the change was insufficient and, for one (residential) facility, just the change in daily practice from the e-learning was insufficient. For (residential) facility 2, the in-person sessions made statistically significantly less change on daily practice. See Table 4 for the ratings.

The actual applications in practice mentioned by the DSPs were: ‘awareness about a healthy lifestyle’ (*n* = 16); a DSP illustrated: ‘*It is in the little things. I am more aware that I let people with ID clean up themselves after dinner instead of doing that for them*’ (respondent 3.33); ‘introducing more physical activities’ (*n* = 14); using the BCTs (*n* = 13); prepare or offer healthy nutrition (*n* = 12); and let people with ID choose themselves (n = 12). Almost all of the DSPs (*n* = 15) indicated nothing has changed in involving family and others in a healthy lifestyle of people with ID. Besides these comments, 11 DSPs had some neutral comments. They indicated the use of the BCTs depends on the people with ID (*n* = 6), and the actual application in practice is dependent on the attitude of the DSP (*n* = 5).

Overall, the managers indicated that the teams spent more attention on a healthy lifestyle in daily practice (see Table 4 for the ratings of the managers). Despite the improvement, the DSPs still struggle with the integration of healthy lifestyle behaviour into working processes.

#### Attitude of DSPs on supporting healthy lifestyle

**Fig. 2:**
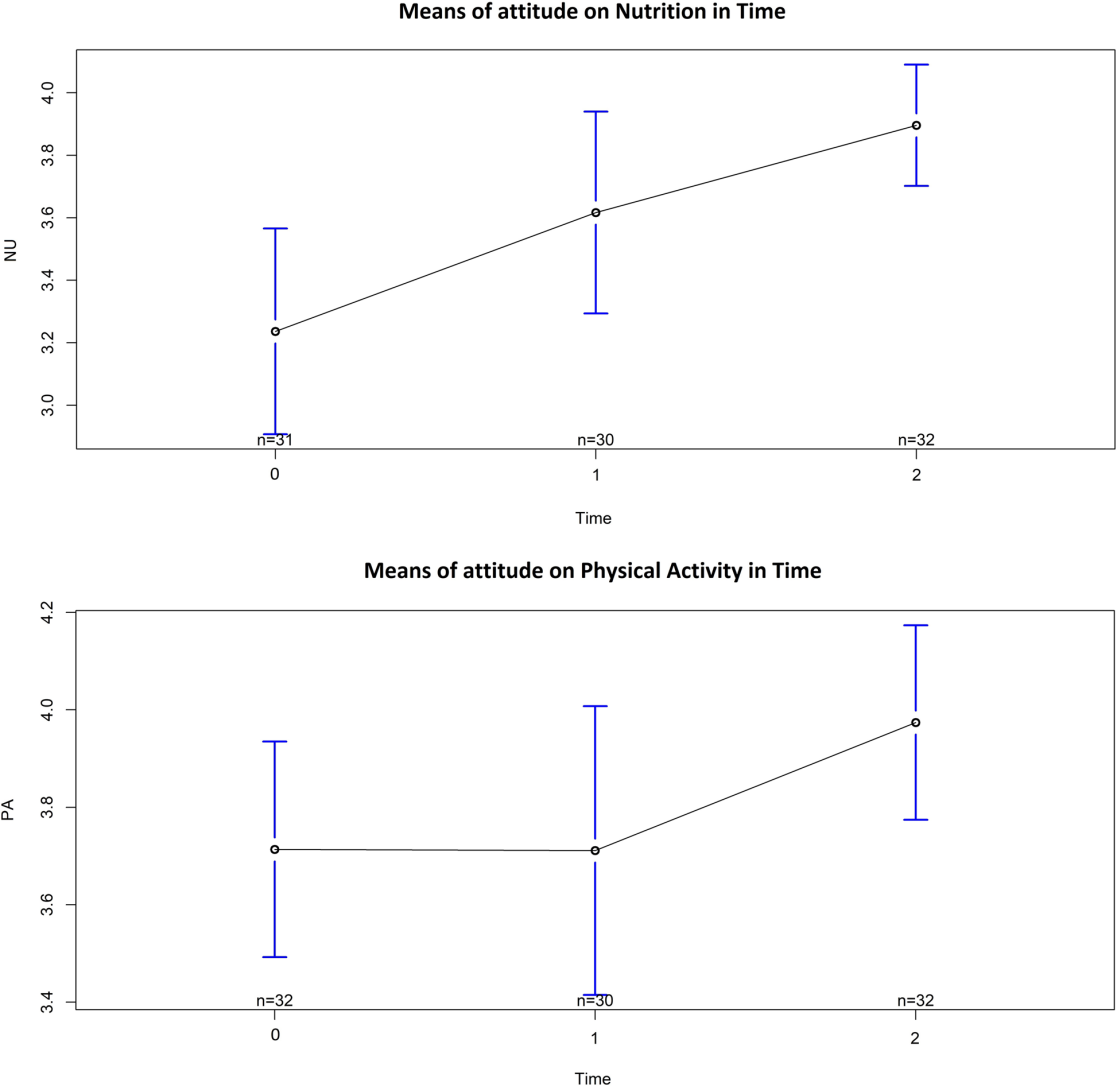
Means of attitude of DSPs on nutrition (NU) and physical activity (PA) at T0, T1, and T2

There is a significant increase over time on the attitudes of nutrition and physical activity of the DSPs between T0 and T2 (3 months after the program), resulting from a mixed model analysis with random effects for DSPs; see Table 5 which is illustrated in Fig. 2. At T1, the change in attitude after the program is not statistically significant for physical activity and, for nutrition, the change is borderline significant.

**Table 5:** Change on attitude of DSPs over time; fixed effects (Estimate) with T0 as reference from mixed modeling using random DSP effects

	Mean	Estimate	Standard error	Degrees of freedom	t-value	*p* < 0.05*
Nutrition intercept (T0)	3.2	3.2	0.1	88.6	23.093	
T1	3.6	0.4	0.2	60.2	1.974	0.05
T2	3.9	0.7	0.2	60.3	3.518	0.00***
Physical activity intercept (T0)	3.7	3.7	0.1	66.3	31.920	
T1	3.7	−0.0	0.1	60.8	−0.023	0.98
T2	4.0	0.3	0.1	60.1	2.112	0.04*

*Significance codes: *** = 0.001, ** = 0.01, * = 0.05

#### Food intake of people with ID

The food intake of people with ID who were supported by the participating DSPs was measured before (T0) and three months after the program (T2). A statistical difference was ascertained between T0 and T2 for food intake from the recommended nutrition guidelines (Z = -1.979, *p* = 0.047). Table 6 shows the descriptives from T0 and T2. For the mean percentages of food intake from the recommended nutrition guidelines, this guideline is not reached.

**Table 6:** Descriptive statistics for percentage food intake from the recommended nutrition guidelines at T0 and T2

	Minimum (%)	Maximum (%)	Median	SD	Z	*p* < 0.05*
T0 (*n* = 22)	30	56	46.00	8.20		
T2 (*n* = 22)	26	75	52.00	12.22	−1.979	0.047*

*Significance codes: *** = 0.001, ** = 0.01, * = 0.05

#### Physical activity of people with ID

The physical activity of people with ID who were supported by the participating DSPs was measured before (T0) and three months after the program (T2). Table 7 shows the mean levels of activity of the people with ID who are able to walk. No significant differences between T0 and T2 were found (% Sedentary: Z = -0.459, *p* = 0.695; % Light: Z = -0.357, *p* = 0.770; % Moderate: Z = -0.357, *p* = 0.750; % Vigorous: Z = -1.604, *p* = 0.250; % Very Vigorous: Z = -1.414, *p* = 0.500). The percentages demonstrate that people with ID spend most of their time in a sedentary state. Table 8 shows the percentages of time spent in activities for people with ID who use a wheelchair; the percentage of time in activities before and after the program is almost the same. During the measurements, two Actigraphs were lost, and one participant did not accept wearing the Actigraph. In addition, the daily activity programs before and after the program were compared. No statistical differences were determined between T0 (Mean: 22.75, SD: 23.77) and T2 (Mean: 24.79, SD: 34.01): Z = -0.280, *p* = 0.844.

**Table 7:** Mean (SD) level of physical activity of walking people with ID (measured by Actigraph)

	% sedentary	% light	% moderate	% Vigorous	% Very Vigorous
T0 (*n* = 14)	64.11 (15.53)	33.25 (14.94)	2.46 (2.70)	0.17 (0.40)	0.01 (0.04)
T2 (*n* = 12)	68.78 (19.08)	28.85 (18.36)	2.29 (2.68)	0.08 (0.24)	0.00 (0.01)
Z	−0.459	−0.357	−0.357	−1.604	−1.414
*p* < 0.05*	0.695	0.770	0.750	0.250	0.500

*Significance codes: *** = 0.001, ** = 0.01, * = 0.05

**Table 8:** Mean (SD) level of physical activity of people with ID using a wheelchair (measured by Actiwatch)

	% inactivity (0–15 counts)	% activity (> 15 counts)
T0 (*n* = 3)	58.67 (29.54)	41.33 (29.54)
T2 (*n* = 3)	58.60 (33.51)	41.40 (33.51)

#### Differences in outcomes for the four participating (residential) facilities

For the differences in outcomes between the four participating (residential) facilities, statistically significant variances were ascertained for the changes after the in-person sessions (actual application in practice)(F = 5.848; *p* = 0.004) and for the relevance (F = 11.606; *p* = 0.000) and satisfaction (F = 14.004; *p* = 0.000) of the in-person sessions (acceptability/suitability). No statistical differences between the four participating (residential) facilities were found for goal achievement, the attitude of DSPs, food intake, and physical activity of people with ID.

## Discussion

### Main findings

The aim of this study was to evaluate the preparation, implementation, and preliminary outcomes of a theory-based training and education program for DSPs to learn how to support people with moderate to profound ID in a healthy lifestyle. The results provide preliminary empirical evidence for this program. Results show that the preparation phase was feasible. Regarding the implementation phase, the overall fidelity of the program was good, although completing the program, the presence, and preparation for the in-person sessions were points of attention. All of the DSPs were positive about the connection of the program to their support needs whereas, for half of the (residential) facilities, the fit of the program was sufficient. The acceptability and suitability of the program were sufficient, however, the face-to-face sessions were rated more positively than the e-learning. The DSPs ranged from slightly negative to positive about the time investment, and the alignment of the mutual expectations of all stakeholders about the program was considered as important and need to be improved. Almost all of the DSPs would recommend the program. The managers were also positive about the content of the program, and the awareness of the importance of a healthy lifestyle in the teams, even though the teams still struggle with the integration of healthy lifestyle behaviour into working processes. Despite the implementation barriers, the DSPs considered the goals of the program to be achieved. The changes after the program were sufficient.

During implementation, barriers were encountered: the DSPs were not always satisfied, the program was not always relevant to them, and they had different experiences about the changes in daily practice after the program. Nevertheless, there were no statistically significant differences in changes after the program between the different (residential) facilities. This may suggest that, despite the mixed opinions of the DSPs regarding the program, it indeed has impact on their attitudes for supporting a healthy lifestyle, which was recognized in a previous implementation study [23]. In this study, the DSPs made positive changes although, in their own perception, they believed their participation in the training program was obligatory.

The DSPs’ attitudes towards supporting a healthy lifestyle on nutrition increased directly after the program. For both nutrition and physical activity, their attitudes significantly increased after 3 months. This is in accordance with previous research in which education was mentioned as a factor that positively influences the attitude of DSPs for supporting physical activity [14, 43, 44]. This improvement of DSPs’ attitudes is important because it contributes to successful implementation of a healthy lifestyle [45, 46].

In addition, for the changes for people with ID after the program, statistically significant improvements were found for food intake; however, for physical activity, no statistical differences were determined. Additionally, the involvement of family and others in a healthy lifestyle for people with ID needs improvement. This social network can be supportive in adapting to the needs of people with ID by focusing on their strengths and, for example, including them in decision-making [47].

### Strengths and limitations

This study was executed in daily practice at four ID care providers; this is where the actual implementation of the program should occur. Although this strength of executing the study in daily practice also brought some limitations for the study, it was the reality of the workplace for the DSPs and therefore provided a realistic representation of the implementation of the program. This study shows important factors that must be taken into account when implementing programs in daily practice.

The changes after the program should be interpreted with caution. The study had a relative small sample size of 32 DSPs and 24 people with ID. In addition, there were missing data in the food diaries of people with ID, and not all persons with ID wore the Actigraphs for the recommended amount of time. Therefore, these outcomes are based on a small sample. However, this is the reality of data collection in daily practice and, nevertheless, the changes after the program were promising.

Although the data collection was suitable for measuring the changes after the program, the feasibility of the data collection process needs improvement regarding the time investment for DSPs. For example, filling in food diaries and wearing physical activity trackers can be components of the daily routine to facilitate the data collection process. The lack of time for implementation corresponds with findings from other implementation studies [12, 23].

Furthermore, physical activity and food intake was measured 3 months after the last program session. This post-test was after the summer holidays, taking into account the absence of regular staff. This may be a limitation because it is questionable if there is a causality between the program and its outcomes, for example, context factors such as changes in the organization, or the different seasons can be a mediating variable. In addition, for future research, a follow-up measurement can indicate healthy lifestyle changes in the long term.

### Implications for implementation

For further implementation of the program, communication and alignment with all stakeholders is an important factor. By discussing mutual expectations in advance, disappointments are avoided, and it improves the relevance of the intervention in daily practice [11]. A contact person who is responsible for managing the mutual expectations and the sustainable execution of the plans would support the actual implementation of the program. The program in this study was flexible to the needs at a specific (residential) facility and adaptable to the target group that DSPs work with; this is an important factor for implementing a health promotion program [22]. Therefore, it is important that at least one of the trainers is familiar with the (residential) facility so that the program can be aligned to the needs of the DSPs. The staff change in teams also affects the knowledge and skills of the team about a healthy lifestyle and impedes the continuation of an intervention [48]. Therefore, it may be considered to perform the program again, maybe in a short form, when hiring new DSPs. In addition, a maximum group size for an intervention should be considered in order to be able to properly conduct the intervention. For larger teams, it can be considered to split them up for optimal utilization.

In the evaluation of the program, for the measurements of people with ID, the residential facility where they live should be responsible for the physical activity and food intake measurements. They are with the people with ID at the beginning and end of the day so are able to check the measurements. This data collection should be incorporated into the daily routine to avoid missing data. The importance of the commitment of staff in the implementation is also addressed in a previous evaluation study [49].

## Conclusions

The theory-based program consisting of a training and education section for DSPs to learn how to support a healthy lifestyle for people with moderate to profound ID was feasible to implement and, despite some barriers regarding time capacity and mutual expectations, it delivered positive changes in both DSPs and persons with moderate to profound ID. Thus, the program is a promising intervention to support DSPs.

## Data Availability

The datasets used and analysed during the current study are available from the corresponding author upon reasonable request.

## Abbreviations

BCTs: Behavior Change Techniques
DIHASID: Discovering Health-promoting Assets in Settings for people with Intellectual Disabilities
DSPs: Direct support professionals
ID: Intellectual disability
IM: Intervention Mapping Protocol
LFE: Low Frequency Filter
NU: Nutrition
PA: Physical Activity
TDF: Theoretical Domains Framework
